# The effect of shape on visual size perception

**DOI:** 10.1177/20416695241270301

**Published:** 2024-08-21

**Authors:** Xiaolin Zhang, Shujie Li, Keli Yin

**Affiliations:** Department of Psychology, 12411 Henan University, Kaifeng, China; School of Public Administration, 12560 Henan University of Economics and Law, Zhengzhou, China; Faculty of Psychology, 26463 Southwest University, Chongqing, China; Department of Psychology, Faculty of Education, 66343 Yunnan Normal University, Kunming, China

**Keywords:** Delboeuf illusion, visual perception, shape, contour attraction, parallel attraction, polygons

## Abstract

The Delboeuf illusion occurs when two circles (test figures) of equal radius are placed side by side and surrounded by concentric circles (inducers) of varying radii, resulting in the test figure being misestimated depending on the size of the surrounding inducer. This study conducted three experiments to explore the impact of shape and the contour attraction and parallel attraction on the Delboeuf illusion for different shapes. In Experiment 1 (*n *= 64), the test figures remained as circles while the inducers varied in shape. Experiment 2 (*n *= 64) involved simultaneous changes in the shape of both the test figures and the inducers. Experiment 3 (*n *= 64) replicated Experiment 2, with the exception that the areas of the inducers were equal and the distances between the inducers and the test figures were also equal. We conclude that the shape of the inducer and the test figure had an impact on the visual size perception, and in the magnitude of the Delboeuf illusion, varied depending on contour attraction. Configurations with circles or shapes resembling circles exhibit contour attraction, while configurations with shapes possessing longer parallel lines shift toward parallel attraction, both attractions enhance the perceived magnitude of the Delboeuf illusion.

Visual information is often ambiguous, which might cause misreading of information in some cases; we call this phenomenon a visual illusion. The Delboeuf illusion is a visual phenomenon introduced by the Belgian philosopher Franz Joseph Delboeuf. In this illusion, when two circles (test figures) of equal radius are presented next to each other and surrounded by concentric circles (inducers) of different radii, the test figure surrounded by a slightly larger inducer is overestimated or underestimated (Murray, 2012; O’Halloran & Weintraub, 1977; Weintraub & Cooper, 1972; Weintraub & Schneck, 1986). The left and right test figures are also called the target and probe, respectively (see Figure 1A). In the Delboeuf illusion, the target's size will be underestimated when the target's inducer (the outer circle) is much larger than the target (the inner circle); in contrast, the target's size will be overestimated when the target's inducer is only slightly larger than the target (Mruczek et al., 2017). In the present study, we explored only the overestimation of the illusion.

**Figure 1.**
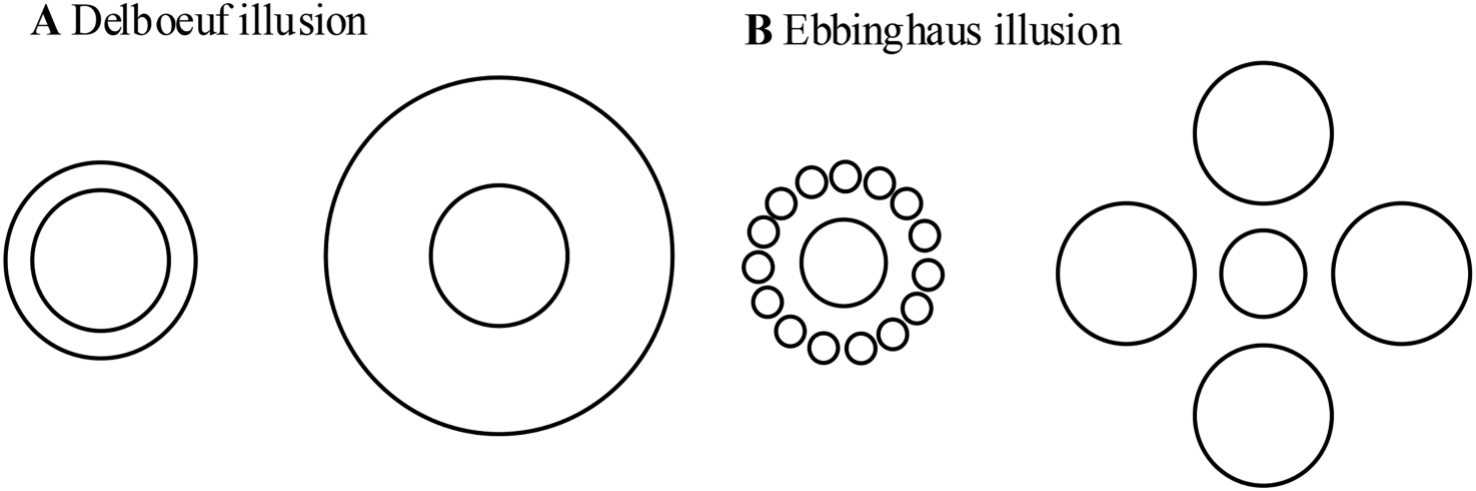
(A) Delboeuf illusion. (B) Ebbinghaus illusion.

Humans’ research on visual illusion may shed light on the evolutionary and environmental influences of perception (Byosiere et al., 2017). The Delboeuf illusion has shown that viewers’ size perception is influenced by luminance (Daneyko et al., 2014) and color (McClain et al., 2014) of the background and stimulus; human research on attribute perceptions of illusion (e.g., shape) is essential, but this avenue has not been sufficiently explored. The shape representations from visual input are crucial to perception, thought, and action (Baker & Kellman, 2018). The perception of many visual illusions requires an overall perception of the target and its surrounding context (Lucon-Xiccato et al., 2019). These findings suggest that research on visual illusions cannot ignore the role of shape.

Humans’ perception of the Delboeuf illusion might be affected by the shape of the test figures or inducers, and further proof is needed (Roberts et al., 2005; Rose & Bressan, 2002; Surkys et al., 2006; Weintraub & Schneck, 1986). Weintraub and Schneck created new models by changing the inducers from a circle to a discontinuous circle, a discontinuous line (four-fragment concentric cases), a square, or several forms around the circle and then compared them with the Delboeuf illusion models of different sizes. They found that the magnitude of the illusion differed when the inducer was a circle or square. Another study by Surkys et al. (2006) changed both the test circles and the inducers’ shape, and they had the same shape. The results showed that the strength of the illusion differed between squares and circles, indicating that shape might influence the illusion's magnitude.

Rose and Bressan (2002) conducted a comprehensive investigation on the Ebbinghaus illusion (see Figure 1B), which exhibits a close relationship with the Delboeuf illusion (Chen et al., 2021; Picon et al., 2019). This study encompassed both same-shape illusions, wherein the test and inducer possessed identical shapes, and different-shape illusions, wherein the test figure constituted a circle while the inducers took the form of circles, hexagons, triangles, or angular shapes. The findings revealed an impact of the inducer shape, as same-shape illusions displayed considerably larger effects compared to different-shape illusions, particularly when circles or triangles served as the test stimuli. Furthermore, the effect of the distance between the test figure and the inducer in the Ebbinghaus illusion shared many commonalities with the Delboeuf illusion. Roberts et al. (2005) attempted to simplify the complexity of influencing factors in the Ebbinghaus illusion and found that the similarity to a circle of the inducer ring was a factor, and smaller inducers constructed more similar to a circle annulus and consistently increased the magnitude of the illusion.

The aforementioned studies primarily pertain to the relationship between shape and perception, with researchers proposing that their findings can be elucidated through the contour attraction. According to this attraction, proximal contours between the test figure and the inducer exert a perceptual interattraction effect, leading to an overestimation of the test figure's size. As the distance between the test and inducer increases, this interattraction diminishes, eventually resulting in perceptual repulsion of distal contours beyond a certain distance. In essence, the magnitude of the Delboeuf illusion is contingent on the distance between the test and the inducer. Within a specific range of distances, the illusion's magnitude intensifies with increasing distance between the test figure and the inducer. However, simultaneously, the strength of the attraction gradually diminishes until it ultimately dissipates. Specifically, when the gap between both circles is relatively small, both circles are perceived as a whole, and individuals holistically pool and assimilate them in the short-term sensory store, leading the test circle to be perceived as larger than it actually is. When the gap between both circles is relatively large and both circles are perceived as two separate percepts, individuals emphasize the differences between them and contrast both circles during the encoding process, leading the test circle to be perceived as smaller than it actually is (Van Ittersum & Wansink, 2012). This attraction phenomenon finds support in other empirical studies (Kawahara et al., 2007; Nakamura et al., 2014), including a functional magnetic resonance imaging study of the visual pathways (Schwarzkopf et al., 2011).

However, Rose and Bressan (2002) proposed that parallel attraction, in addition to contour attraction, is a significant factor in explaining the Ebbinghaus illusion. They thought that the concentric circles of Delboeuf illusion, in which “adjacent” contours can be said to run in parallel, could also influence the Delboeuf illusion involving polygons. Pollack (1964) found that attraction between nonintersecting contours was maximal when they were parallel, and the attraction gradually decreased as the angle of the projected intersection of boundaries increased; this phenomenon was named parallel attraction.

Moreover, Pollack (1964) delved into the examination of the influence of utilizing arcs to confine straight boundaries, yielding insightful findings that indicate the distinctive nature of arcs as boundary elements. In the scenario of a closed component comprising numerous arcs (a circle is the most special case), wherein infinitesimally short tangents are drawn to adjacent points along the circumferences of the arcs, these tangents, extending across space, exhibit a parallel relationship to the tangents of corresponding points on the other arc. The visual system processes a circular stimulus as a group of straight lines (Ito, 2012), and there are numerous orientation-tuned cells in area V1 of the cortex (Hubel & Wiesel, 1959). The receptive field of the orientation-tuned cells in peripheral vision is considered to be relatively large; therefore, a rough approximation of a circle's curvature produced by a small number of orientation-tuned cells or a limited number of orientationtuned channels (Ellemberg et al., 2006) might become visible after adaptation to curves. Consequently, the closed component exemplifies a state of near-maximal parallelism, thereby consistently generating an attractive effect that intensifies as the arcs become more flattened. It is as if the contour attraction transforms into parallel attraction, representing a shift in effect or a gradual change in form.

Therefore, we maintain that contours consistently exist, while parallel lines are observed in specific circumstances. Such conditions perpetuate potential interactions between the contours of a central shape and those of its surrounding elements, consequently leading to the perception of parallel attraction. We hypothesize that the Delboeuf illusion demonstrates both contour attraction across various shape variations and parallel attraction effects in certain configurations. We postulate that in the classic Delboeuf illusion, contour attraction is in the dominant situation, leading to a stronger illusion magnitude in shapes that are circular or closely resemble circles, whereas for shapes with sufficiently elongated parallel lines, parallel attraction is in the dominant situation, parallel attraction becomes salient and the shapes with elongated parallel lines get stronger illusion magnitude, resulting in different perceptual effects arising from these two forms of attraction. In essence, the two types of attraction appear to represent a continuum of effects, when shapes are very similar to circles, contour attraction becomes perceptual, whereas substantial deviations from circularity, coupled with pronounced flattening of the parallel lines, make parallel attraction perceptual.

Furthermore, prior investigations (Roberts et al., 2005; Rose & Bressan, 2002; Surkys et al., 2006; Weintraub & Schneck, 1986) have identified the necessity for enhancing the experimental methodologies concerning the Delboeuf illusion. In our current study, we adopted a quasi-Delboeuf configuration, wherein regular polygons were utilized as substitutes for the traditional circular test figures and inducers in the classical Delboeuf illusion configuration. Specifically, the inducers (circle) are used as circumscribed circles to control the size of the regular polygons while keeping the test figure circle, the test figures and the inducers are used as circumscribed circles to create a regular polygon configuration, and the test figure and the inducers are used as inscribed circles to ensure that the distances of the test figure and the inducer are equal in the regular polygon configuration (for more details, see Appendix). The inner polygon was referred to as the test figure, and the outer polygon was referred to as the inducer. The radius of the polygon was measured as its circumscribed/inscribed circle.

We aimed to systematically modulate the shape of the test figure and inducer. We selected the following shapes as independent variables: circle, regular octagon, regular hexagon, square, and equilateral triangle. By employing these shapes, we establish five distinct levels ranging from a circle to a triangle, with each level progressively deviating further from a circular form. These shapes are perceived as distinct figures; however, they are equivalent in the present quasi-Delboeuf illusion configurations from the viewpoint of topology (Chen, 1982). This series of shapes creates the condition in which the contour attraction and the parallel attraction by the shape can be examined in the Delboeuf illusion. Furthermore, the utilization of the quasi-Delboeuf illusion configuration provides an opportunity to standardize the classification of configurations pertinent to this illusion. This approach ensures greater consistency and comparability in our experimental investigations.

We designed two distinct types of shape-changing scenarios for Experiments 1 and 2. In Experiment 1, we maintained a consistent circular shape for the test figure across all experimental conditions while solely altering the shapes of the inducers. The purpose of this experiment was to investigate the influence of shape and contour attraction in the Delboeuf illusion when parallel lines were not present in the configurations. Conversely, in Experiment 2, we introduced simultaneous changes in the shapes of both the test figure and the inducer in different conditions, leading to the formation of parallel lines within the illusion configurations. By conducting Experiments 1 and 2, we aimed to examine the impact of shape variations in both scenarios, and the comparative analysis between the two experiments would unveil the specific effects of parallel attraction in the Delboeuf illusion.

In Experiments 1 and 2, the areas of the inducer and the distance between the test figure and the inducer decreased from a circle to an equilateral triangle, which would have unnecessary impacts on the size estimation. It is necessary to maintain an equal area and distance between the target stimulus and the inducer in varied shapes. In Experiment 3, the shape of the target stimulus (the test figures on the left) and the inducer changed simultaneously while their areas and the distances between the target stimulus and the inducer were equal. A comparative analysis between Experiments 2 and 3 would elucidate that in the absence of factors such as area and distance , the parallel attraction would affect the illusion. It should be noted that the parallel attraction would be evident in the shape with enough parallel lines in Experiments 2 and 3. Therefore, the effect would be evident in the shapes most dissimilar to a circle: the square and the equilateral triangle, or at least the equilateral triangle. Therefore, the first hypothesis was as follows:

The illusion magnitude would significantly differ by shape, and the magnitude of the illusion in different shapes would be different.

The second hypothesis was as follows:

The illusion magnitude of the polygons in Experiment 2 would be significantly larger than the identical shape in Experiment 1. The more similar to a circle the polygon shapes were, the less difference there would be in quantity.

The third hypothesis was as follows:

The illusion magnitude of at least the equilateral triangle in Experiment 3 would be significantly larger than that in Experiment 2.

In the present study, we explored whether parallel attraction or contour attraction holds for inducers of varying shapes using polygons and how the shape of the test figures and inducers in a Delboeuf illusion configuration influenced the illusion magnitude. Accordingly, we explored the effect of shape on the magnitude of the Delboeuf illusion and the effect of contour attraction and parallel attraction in the Delboeuf illusion.

## Method

### Experiment 1

#### Participants

Sixty-four undergraduates (32 female; mean age = 20.53 years, *SD *= 1.50 years) volunteered to participate in the experiments and had normal or corrected-to-normal vision. All were right-handed and were unfamiliar with or unaware of the Delboeuf illusion. All participants provided informed consent and were given a gift (equivalent to approximately US$6) for their participation. The study design conformed to relevant ethical statements and was approved by the academic council.

#### Materials and Procedure

Stimuli were displayed on a 19-inch LCD monitor (Model 943NW, Samsung, Huizhou, China) with a resolution of 1440 × 900 pixels. The black (RGB 0, 0, 0) configurations were displayed and manipulated with Geometer's Sketchpad 5.03 on a white (RGB 255, 255, 255) background. The line weight was 0.5 points. Participants were in a bright and comfortable laboratory, seated and instructed to maintain their gaze at a prescribed distance of approximately 60 cm from the computer, with their head orientation stabilized by a support device, during the iterative process of creating a novel configuration for the probe stimulus. In all three experiments, the spatial offset between the central points of the test figure and the target was kept at 15°. On the left side, the radii of the targets and inducers were 2.5° and 3.75°, respectively, while on the right side the inducers had radii of 6.25° in Experiments 1 and 2. In Experiment 3, all polygonal inducers exceeded a radius of 6.25°. The ratios between the radii of the test figures and the inducers ranged from 2:3 to 2:5, a range previously identified as indicative of overestimation in the quasi-Delboeuf illusion paradigm (Mruczek et al., 2017). We tried our best to keep overestimating the magnitude of the quasi-Delboeuf illusion.

We adopted a repeated-measures, single-factor, within-subject design. The independent variable was the shape of the configuration: circle, regular octagon, regular hexagon, regular quadrilateral (square), and equilateral triangle. The order of trials was counterbalanced using a Latin square design. The first order of shape was circle (A), regular octagon (B), regular hexagon (C), square (D), and equilateral triangle (E). 

**Figure 2.**
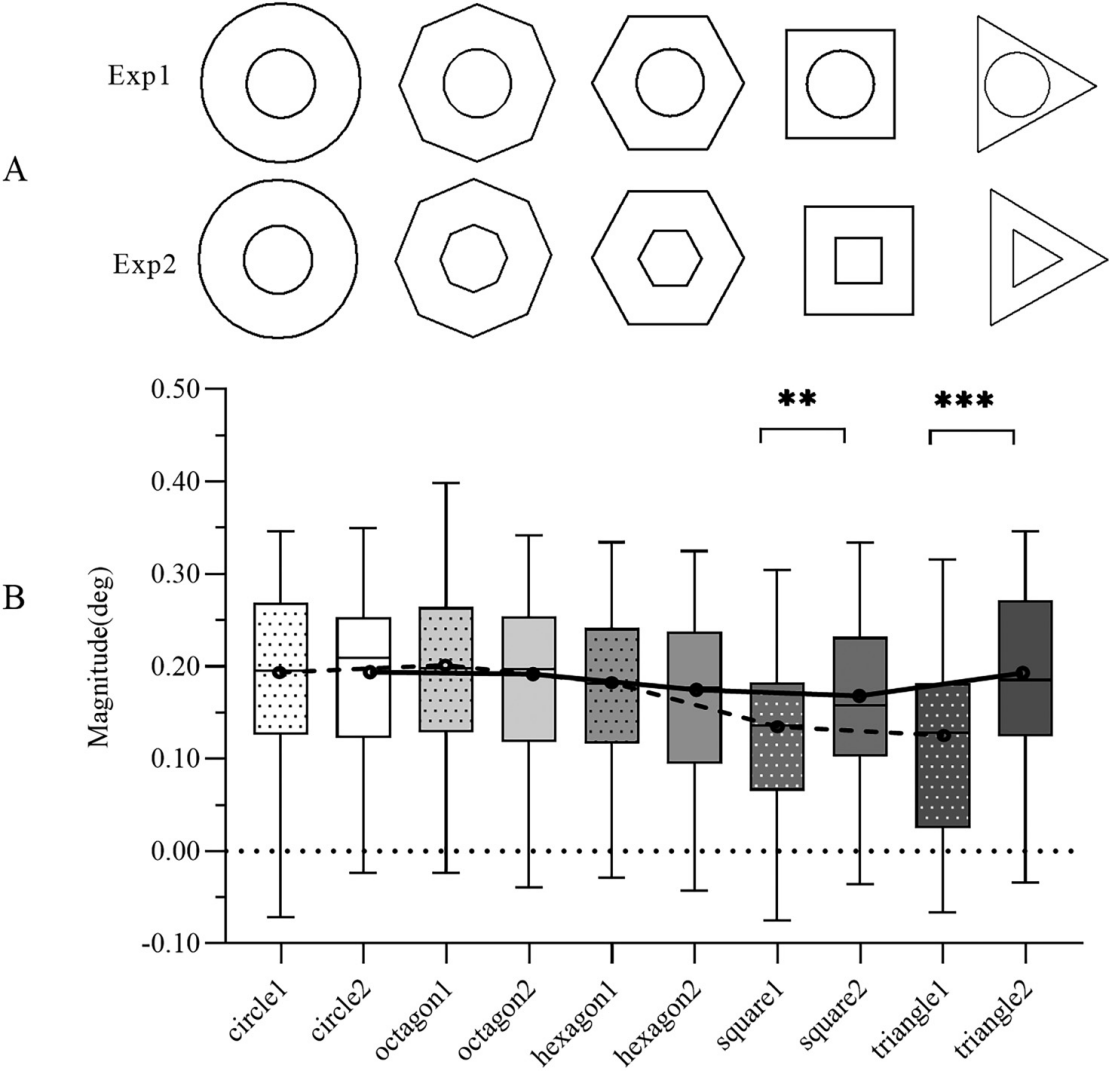
(A) The configuration of the illusion in Experiments 1 and 2. (B) A comparative analysis of magnitudes between Experiments 1 and 2. The results from Experiment 1 are indicated with a dashed color fill and are labeled with the corresponding shape followed by “1.” Similarly, the results from Experiment 2 are denoted by a solid color fill and are labeled with the respective shape followed by “2.” Paired samples testing revealed significant differences in the magnitudes of the circle, square, and equilateral triangle between Experiments 1 and 2. The trendline depicting the illusion magnitude for each shape is displayed as a dashed line for Experiment 1 and a solid line for Experiment 2. In the boxplots, median values are represented by horizontal lines, while the length of the boxes illustrates the interquartile range. The error bars denote the upper and lower bounds of the 95% confidence interval.

**Figure 3.**
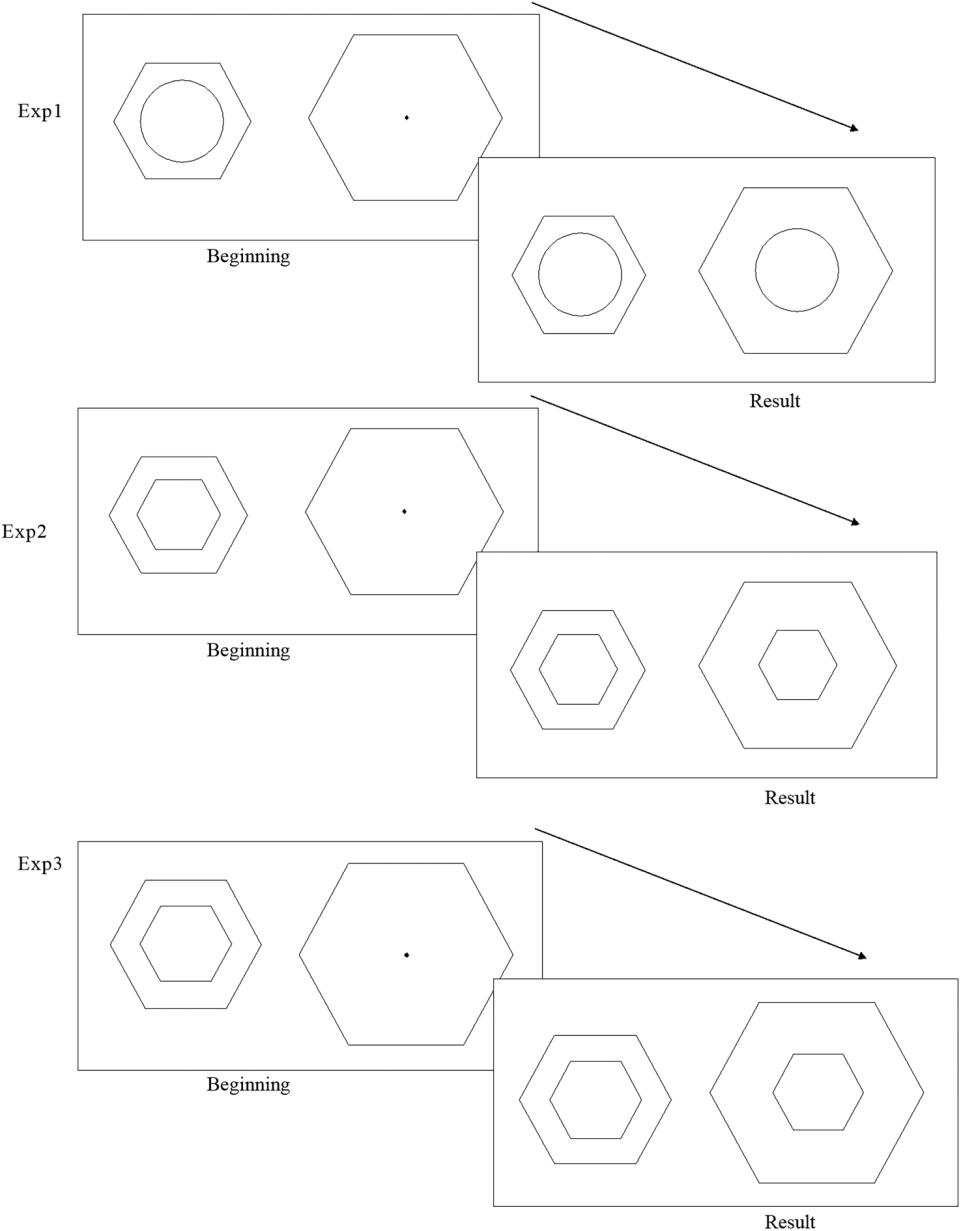
In each trial, a target stimulus was presented alongside an inducer on the left side of the screen. On the right side, an inducer was displayed without a probe stimulus. Participants were instructed to generate a probe stimulus by clicking on the central point of the right larger inducer and subsequently adjusting its size by dragging the mouse until they perceived it to be equal to the target stimulus.

The procedures were tested in a pilot experiment, with a break between each experiment. In each experiment, participants completed a total of 75 trials, with 15 trials per shape. Throughout the primary experiment, participants were tasked with focusing their attention on the designated target stimulus. Their objective was to modify the dimensions of the newly introduced probe stimulus based on their initial perceptions. At the commencement of each trial, a target stimulus (a circle in Experiment 1) was exhibited on the left side of the screen, encircled by either a circle or an alternate regular polygon. Positioned on the right side was a sizable circle or a large regular polygon (referred to as the inducer stimulus). Notably, this right-sided stimulus lacked the inclusion of the test figure (the probe stimulus). Participants were then instructed to fashion a new pattern for the probe stimulus, aligning it with the central point of the larger inducer on the right. Accomplishing this involved the adjustment of the probe stimulus's size through mouse dragging until they judged it to be equivalent to the target stimulus. There was no time limit so they could adjust the size as they wished, and they were allowed to make multiple minor adjustments (which could solve the problem of the number of trials being small to some extent). Figure 2 shows the procedure for one trial in each experiment. The target stimulus, probe, and inducers shared the same shape in each trial (see Figure 3A), and the presentation order of experiments was identical between the different participants.

The radius of the designated target stimulus was denoted as “r,” and that of the newly created probe stimulus was represented as “R,” with both measurements quantified in degrees of visual angle. The magnitude of the illusion was R–r. This difference value in radii between the probe stimulus and the target stimulus served as a metric to determine the magnitude of the illusion. The measurement that we used for the radii of the configurations as illusion magnitude was consistent with that described in past research on size illusions (O’Halloran & Weintraub, 1977; Weintraub & Cooper, 1972; Weintraub & Schneck, 1986).

In our experimental design, we employed a set of five distinct polygons exhibiting varying degrees of resemblance to a circle. This systematic manipulation of shapes represents an advancement over previous research, which typically focused on only two shapes (circle and square) to explore the magnitude of the quasi-Delboeuf illusion (Surkys et al., 2006). Additionally, we adopted a novel approach in which participants were tasked with creating a probe stimulus based on their own will rather than selecting from a predefined list of forms. This method ensured that the measured magnitude of the illusion corresponded to the participants’ subjective perceptions, thereby utilizing the subjective equality point as the measurement outcome. In Experiment 1, the test figure remained consistent as a circle throughout all the various conditions, while the inducers changed different conditions.

#### Results

The average values of the magnitude of the illusion for each shape were calculated and used in the analyses (see Figure 3B). The magnitude of the illusion differed by shape from circle to equilateral triangle (except for the regular octagon), the magnitude of the illusion decreased, and the trendline of the illusion magnitude for each shape is represented by a dotted line in Figure 3B.

Mauchly's test of sphericity indicated that the data did not meet the assumptions of the applied tests, *W *= .666, *p *= .003. The general linear model (GLM) repeated measures was adjusted using the Greenhouse‒Geisser correction and showed that the illusion magnitude was significantly dependent on the shape, *F*(3.358, 211.129) = 32.129, *p *< .001, η*_p_*^2^*^ ^* = .338. Post hoc tests, adjusted using the Sidak correction, indicated significant differences for all two-shape comparisons (*p*s* < *.05), with the exceptions of the circle with the regular octagon and regular hexagon and the square with the equilateral triangle. The mean and the 95% confidence interval for each shape of the illusion magnitude are shown in Table 1. The details for the comparison of magnitude can be seen in the dotted color fills in Figure 3B.

**Table 1.** The magnitude of each shape in the three experiments.

Shape	Exp1 *n *= 64			Exp2 *n *= 64			Exp3 *n *= 64		
	*M*	95% CI		*M*	95% CI		*M*	95% CI	
		LB	UB		LB	UB		LB	UB
Circle	.190	.167	.213	.186	.164	.208	.192	.172	.212
Octagon	.199	.177	.220	.184	.159	.209	.195	.173	.217
Hexagon	.177	.155	.198	.166	.143	.190	.151	.125	.176
Square	.121	.098	.144	.159	.137	.182	.190	.171	.208
Triangle	.110	.086	.134	.185	.159	.212	.237	.213	.260

### Experiment 2

The primary objective of Experiment 1 was to explore the influence of shape and contour attraction in the Delboeuf illusion configurations without the presence of parallel lines. In contrast, Experiment 2 was designed to introduce simultaneous changes in the shapes of both the test figure and the inducer under various conditions, resulting in the formation of parallel lines within the illusion configurations. Conducting Experiments 1 and 2 allowed us to investigate the impact of shape variations in both scenarios.

#### Participants and Procedure

Following a brief intermission of five minutes, participants commenced Experiment 2. The participants, methodology, and procedural steps remained consistent with those employed in Experiment 1, with the sole exception being the concurrent alteration of both the test figure and the inducer shapes (depicted in Figure 3A). The study design conformed to relevant ethical statements and was approved by the academic council.

#### Results

The average values of the magnitude of the illusion for each shape were calculated and analyzed by PASW Statistics 20. The data showed that the magnitude of the illusion is dependent on the shape (see Figure 3B).

The significance threshold applied to our data was *p *< .05. Mauchly's test of sphericity indicated that the data did not meet the assumptions of the applied tests, *W *= .744, *p *= .033. The GLM repeated measures was adjusted using the Greenhouse‒Geisser correction and showed that illusion magnitude was significantly dependent on shape, *F*(3.523, 221.967) = 3.605, *p *= .010, η*_p_*^2^*^ ^* = .054. Post hoc tests, employing the Sidak correction for adjustments, revealed nonsignificant differences among the compared shapes. However, the use of the least significant difference test indicated statistically significant differences between the circle and the regular hexagon (*p = *.048) and the square (*p = *.005) and between the square and the regular octagon (*p *= .013) and equilateral triangle (*p = *.005). The shape had an impact on the magnitude of the illusion of the regular octagon, square, and equilateral triangle in this experiment. From the regular octagon to the equilateral triangle, the illusion magnitude of the square was minimal (inflection point). The trendline of the illusion magnitude for each shape is represented by a solid line in Figure 3B. The mean and the 95% confidence interval for each shape of the illusion magnitude are shown in Table 1, and the details for the comparison of magnitude can be seen in the solid color fills in Figure 3B.

Nevertheless, the outcomes did not elucidate additional influential factors. The experimental configurations in study 1 exhibited essential elements of both contour attraction and, to some extent, parallel attraction. Nonetheless, distinguishing the specific effects remained a challenge. To delve more profoundly into the effect of parallel attraction, a thorough investigation was undertaken to determine whether the magnitude of the illusion was influenced by this phenomenon, particularly in situations where the polygons exclusively featured parallel lines.

#### Comparison of the Results of Experiments 1 and 2

Comparing the outcomes of Experiments 1 and 2 provides valuable insights into the combined influence of parallel lines and shape on the magnitude of the Delboeuf illusion. Our findings reveal that the illusion magnitude is notably affected by the shape, both when the shapes of the test figures and the inducers are changed simultaneously (Experiment 2) and when only the shape of the inducers is altered while keeping the test figure as a circle (Experiment 1). These observations underscore the significance of shape in influencing the magnitude of the quasi-Delboeuf illusion. Further analysis of Experiments 1 and 2 revealed that the highest magnitude of the illusion was observed for the regular octagon and equilateral triangle, respectively. In the latter experiment, the magnitude gradually declined from the regular octagon to the square. A comparison of the magnitude between Experiments 1 and 2 is shown in Figure 3B.

Mauchly's test of sphericity indicated that the data did not meet the assumptions of the applied tests, *W *= .787, *p *< .001. The GLM repeated measures comparing the results of Experiments 1 and 2 was adjusted using the Greenhouse‒Geisser correction and confirmed that inducer shape significantly influenced illusion magnitude, *F*(3.597, 453.193) = 22.622, *p *< .001, η*_p_*^2^*^ ^* = .152. Furthermore, there was a nonsignificant effect between the two experiments, *F*(1, 126) = 1.465, *p = *.228. The interaction between the shape and the test figure was significant, *F*(3.597, 453.193) = 16.047, *p *< .001, η*_p_*^2^*^ ^* = .113.

A paired samples test applying a Bonferroni correction and since there were five factors, the significance threshold applied to the data was *p *< .01. The results showed that in Experiments 1 and 2, the magnitudes of the square, *t*(63) = −3.512, *p *= .001, and the equilateral triangle, *t*(63) = −5.970, *p *< .001, were significantly different. The mean and the 95% confidence interval for each shape of the illusion magnitude are shown in Table 1. For more details on the comparisons between Experiments 1 and 2, see Figure 3B.

In both Experiments 1 and 2, the test figures and inducers that comprised the quasi-Delboeuf illusion took the form of polygons, with their radii being equivalent to those of circles. As a result, the distances between the test figures and inducers decreased as one transitioned from circles to equilateral triangles. This leads us to the question: would the magnitude of the Delboeuf illusion be impacted by the distance between the test figures and the inducers or by the shape of configurations? We could maintain an equal distance between the test figure and inducer in varied shapes and maintain equal areas of all the probes’ inducers and targets’ inducers.

### Experiment 3

#### Participants and Procedure

**Figure 4.**
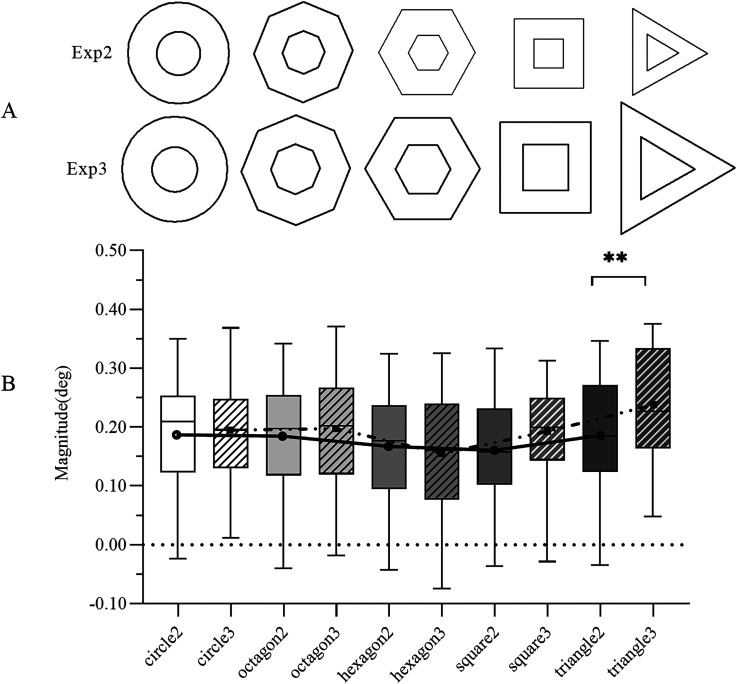
(A) The configuration of the illusion in Experiments 2 and 3 reveals a notable distinction: the radii of the polygonal inducers’ circumcircles in Experiment 3 surpass those observed in Experiment 2. (B) A comparative analysis of magnitudes between Experiments 2 and 3. The results from Experiment 2 are indicated with solid color fill and are labeled with the corresponding shape followed by “2.” Similarly, the results from Experiment 3 are denoted by a diagonal color fill and are labeled with the respective shape followed by “3.” Paired samples testing revealed significant differences in the magnitudes of the equilateral triangle between Experiments 2 and 3. The trendline depicting the illusion magnitude for each shape is displayed as a solid line for Experiment 2 and a dotted line for Experiment 3. In the boxplots, median values are represented by horizontal lines, while the length of the boxes illustrates the interquartile range. The error bars denote the upper and lower bounds of the 95% confidence interval.

Following a brief intermission of 5 min, participants commenced Experiment 3. The participants, research methodology, and procedural protocol mirrored those implemented in Experiment 2, with the exception that the areas of all the inducers were equal, and all the distances between the inducers and test figures were also equal (depicted in Figure 4A). The study design conformed to relevant ethical statements and was approved by the academic council.

#### Results

Average values for the illusion magnitude of each shape were calculated and used in the analyses. The magnitudes followed the same tendency as in Experiment 2 but with higher absolute values in the square and the equilateral triangle (see Figure 4B).

Mauchly's test of sphericity indicated that the data did not meet the assumptions of the applied tests, *W *= .756, *p *= .047. The GLM repeated measures was adjusted using the Greenhouse‒Geisser correction and showed that the illusion magnitude was significantly dependent on the shape, *F*(3.501, 220.594) = 18.708, *p *< .001, η*_p_*^2^*^ ^* = .229. Post hoc tests, adjusted using the Sidak correction, indicated significant differences for all two-shape comparisons (*p*s* < *.01), with the exceptions of the circle with the regular octagon and the square, the regular octagon with the square, and the regular octagon with the equilateral triangle. From the regular octagon to the equilateral triangle, the illusion magnitude of the regular hexagon was minimal (inflection point). The trendline of the illusion magnitude for each shape is represented by a dotted line in Figure 3B. The mean and the 95% confidence interval for different shapes of illusion magnitude are shown in Table 1, and the details for the comparison of magnitude can be seen in the diagonal color fills in Figure 4B.

#### Comparison of the Results of Experiments 2 and 3

The results of Experiments 2 and 3 showed that the magnitude of the quasi-Delboeuf illusion changed with the shape when the test figure and the inducers changed simultaneously (Experiment 2), as well as when the areas of the inducers and the distances were equal (Experiment 3). A comparison of Experiments 2 and 3 is shown in Figure 4B.

Mauchly's test of sphericity indicated that the data did not meet the assumptions of the applied tests, *W *= .815, *p *= .003. The GLM repeated measures was adjusted using the Greenhouse‒Geisser correction and showed that the illusion magnitude was significantly dependent on the shape, *F*(3.603, 454.005) = 16.451, *p *< .001, η*_p_*^2^*^ ^* = .115. There was a nonsignificant effect between the two experiments, *F*(1, 126) = 1.423, *p *= .235. The interaction effect of shape and area of the inducers was significant, *F*(3.603, 454.005) = 7.044, *p *< .001, η*_p_*^2^*^ ^* = .053.

A paired samples test applying a Bonferroni correction and since there were five factors, the significance threshold applied to the data was *p *< .01. The results showed that in Experiments 2 and 3, the magnitude of the illusion of the equilateral triangle was significantly different between Experiments 2 and 3, *t*(63) = −2.876, *p *= .005. For more details on the comparisons between Experiments 2 and 3, see Figure 4B.

#### Comparison of the Results of Experiments 1 and 3

Comparison of the results of Experiments 1 and 3 would evaluate the individual correlation between the performances in the two experiments.

Mauchly's test of sphericity indicated that the data did not meet the assumptions of the applied tests, *W *= .792, *p *< .000. A GLM repeated measures was adjusted using the Greenhouse‒Geisser correction and showed that the illusion magnitude was significantly dependent on the shape, *F*(3.598, 453.339) = 12.177, *p *< .001, η*_p_*^2^*^ ^* = .088. There was significant effect in the two experiments, *F*(1, 126) = .6.654, *p *= .011, η*_p_*^2^*^ ^* = .050. The interaction effect of the shape and the experiments was significant, *F*(3.598, 453.339) = 33.994, *p *< .001, η*_p_*^2^*^ ^* = .0236.

A paired samples test applying a Bonferroni correction and since there were five factors, the significance threshold applied to the data was *p* < .001. The results showed that in Experiments 1 and 3, the magnitude of the illusion of the square, *t*(63) = −4.830, *p *< .001, and the equilateral triangle, *t*(63) = −7.773, *p *< .001, were significantly different between Experiments 1 and 3.

## Discussion

We investigated the influence of shape on the magnitude of the Delboeuf illusion, with a particular focus on its implications for contour attraction and parallel attraction. Our study builds on previous research by using three different conditions: (1) where the shape of the inducers varied while the test figures remained circular, (2) where the test figures and inducers varied in identical shapes, resulting in the formation of parallel lines, and (3) where the shape of the test figures and inducers varied in identical shapes while maintaining equal areas and distances between the target stimulus and the inducers. Each of these conditions serves to elucidate the impact of shape and unveil the relationship between contour attraction and parallel attraction within the Delboeuf illusion.

In our experimental design, we employed five distinct types of polygons, each exhibiting varying degrees of similarity to a circle: the regular octagon, regular hexagon, square, equilateral triangle, and circle. This diversified approach contrasts with previous investigations (Surkys et al., 2006) into the magnitude of the quasi-Delboeuf illusion, which primarily focused on two shapes, namely the circle and square. Participants were instructed to generate a probe stimulus based on their own subjective judgment rather than selecting from a predefined set of forms. This methodological refinement aimed to enhance the precision of measuring the illusion's magnitude by aligning it with participants’ subjective perceptions rather than relying on a predetermined selection criteria. By implementing this approach, we effectively mitigated the issue of discontinuous data presentation observed in prior research endeavors (e.g., Surkys et al., 2006), thereby ensuring that the measured magnitude of the illusion matched the participants’ continuous subjective perception.

In addition, our experiments introduced the notion of a quasi-Delboeuf illusion configuration, facilitating the standardization of configuration classification for this illusion. The methodology of using configuration radii to measure illusion size is consistent with previous research on size illusions (O’Halloran & Weintraub, 1977; Weintraub & Cooper, 1972; Weintraub & Schneck, 1986). Specifically, in our experiments, the ratios of the radii of the test figures to the inducers were between 2:3 and 2:5, a known range associated with overestimation in the quasi-Delboeuf illusion. This ensured that overestimation was as prevalent as possible, rather than contrast, as the dominant effect in our experiments.

### Impact of the Inducer Shape on Illusion Magnitude

Experiment 1, involving the modification of only the inducer shape, and Experiments 2 and 3, wherein both the test figures and inducers were altered concurrently, revealed significant variations in the magnitude of the illusion across different shapes. Both the test figure and inducer shape exhibited substantial effects on the illusion magnitude, with the inducer shape demonstrating a particularly pronounced influence, as evident from the outcomes of Experiments 1 and 2. Remarkably, even in Experiment 3, where configurations were matched in terms of area and the distances between the test figure and the inducer, the shape persisted as a critical factor impacting the illusion magnitude. Hence, our findings underscore the dominant role played by the inducer and the test figure shape in determining the magnitude of the quasi-Delboeuf illusion, thus corroborating the significance of object context, as proposed by Bar (2004).

### Contour Attraction is Dominant

Contour attraction postulates that the spatial separation between the inducers and the test figure is a critical factor influencing the illusion magnitude. Within a certain range, the magnitude of the illusion increases as the distance between the inducers and the test figure increases. However, beyond a certain distance threshold, the perceptual interattraction between the test figure and the inducers weakens and eventually dissipates. In Experiment 1, where the test figure consistently maintained a circular shape, the distances between the test figure and the inducers gradually decreased as the inducers transitioned from a regular octagon to an equilateral triangle. Concurrently, as the distances diminished, the illusion magnitudes also decreased accordingly. This result provides empirical support for the validity of the contour attraction phenomenon.

Furthermore, the contour attraction can also elucidate the observed pattern of similarity effects relative to a circle. The more similar the shapes of the inducer and the test are, could form the most ideal attraction conditions, the easier it is to perceive them as a whole (McClain et al., 2014), shapes that bear a closer resemblance to a circle are associated with larger proximal contours, leading to a stronger attraction effect. The regular octagon, being more similar to a circle than the regular hexagon, and the regular hexagon, being more akin to a circle than a square, experienced a gradual decrease in the magnitude of the illusion. This interpretation effectively accounts for the data obtained from Experiment 1 (excluding the circle), reinforcing the role of contour attraction in shaping the illusionary effects based on the similarity to a circular form. Consequently, the circle or the regular octagon was the most substantial overestimation of the target stimulus size. Our empirical results align well with this expectation.

Certainly, while a circle is inherently the most similar shape to itself, the configuration involving circles did not yield the highest magnitude in our experimental outcomes, presenting an incongruity with the principles of contour attraction. According to the contour attraction hypothesis, when both the test figure and the inducers are circles, the illusion magnitude should reach its maximum. However, the observed inconsistency may be attributed to the remarkable perceptual acuity of humans in discerning and identifying slight deviations from the perfect circular form (Wilkinson et al., 1998). Consequently, the size of the test figure placed on circles would more closely match the participants’ perceptions, resulting in a relatively smaller magnitude of the quasi-Delboeuf illusion for circles compared to regular octagons or regular hexagons. Based on this elucidation, it is clear that our results support contour attraction.

Through comparative analysis between Experiments 1, 2, and 3, the results indicate variability in the magnitude of the illusion experienced with circle, octagon, and hexagon shapes within the same experimental condition due to shape changes. However, no statistically significant disparities were observed between Experiments 1 and 2, nor between Experiments 2 and 3. This indicates that no discernible distinctions were found across the various experimental conditions. The variance noted between Experiments 1 and 3 primarily arises from the impact of distance, which is a constituent element of contour attraction. This result emphasizes the persistent impact of contour attraction on shape perception across all three experimental conditions.

### Parallel Attraction as a Distinct Form of Contour Attraction

In Experiment 2, the test figures changed from circles to equilateral triangles, resulting in a gradual decrease in the distances between the inducers and the test figures. As a result, one would anticipate a reduction in the magnitude of the illusion. However, the anticipated decline in illusion magnitude did not occur for the square and equilateral triangle. It is possible that contour attraction phenomena are observed in these polygonal shapes and accompanied by parallel attraction effects, which would increase rather than decrease the magnitude of the illusion.

In Experiment 2, the test figures and inducers formed parallel lines (sides) for the square and the equilateral triangle, whereas this parallel arrangement was absent in Experiment 1. The illusion magnitude for the square and the equilateral triangle surpassed that of Experiment 1 due to the evident emergence of the parallel attraction effect. Notably, the equilateral triangle exhibited three pairs of parallel lines (sides), while the square had four such pairs (see Figure 3A). Additionally, the parallel lines in the equilateral triangle were the longest among the four polygonal shapes, followed by the square. As the square and the equilateral triangle possessed longer parallel lines, the parallel attraction effect became more pronounced. Consequently, the equilateral triangle exhibited the strongest parallel attraction, followed by the square, and this trend extended to Experiment 3 as well. We can therefore deduce that the parallel attraction gradually increases as shapes move from circles to regular polygons, or from circle-like shapes to squares or equilateral triangles. In these configurations, all shapes are characterized by parallel lines and differ significantly from a circular shape.

Furthermore, concerning the regular hexagon and the regular octagon, no statistically significant differences were observed between Experiments 1 and 2, nor between Experiments 2 and 3, indicating that the effects of contour attraction (in Experiment 1) and parallel attraction (in Experiment 2) did not exhibit significant divergence. In particular, in the context of the classic Delboeuf illusion, the “adjacent” contours are assumed to be parallel (Rose & Bressan, 2002), implying the presence of a parallel attraction that can subsequently transform into a contour attraction. It is conceivable that a transformation of shapes may engender a transition from one form of attraction to another.

While the gradual transformation from a circle to a triangle presents a realistic progression, delineating the boundary or distinction between contour attraction and parallel attraction proves elusive. These phenomena do not denote different types of attraction; rather, they manifest as different expressions of a singular underlying phenomenon, with parallel attraction being essentially a manifestation of contour attraction. In specific circumstances, both forms of attraction may concurrently operate, whereas in other situations, one form may transition into the other. Notably, when shapes closely approximate a circle, the interaction between the test stimuli and the inducers predominantly manifests as contour attraction. Conversely, shapes characterized by elongated parallel lines tend to transform the interaction to parallel attraction.

Intrinsic contour attraction between a central geometric form and its surrounding counterpart is a pervasive phenomenon across diverse geometries, while the phenomenon of parallel attraction manifests under specific conditions. The gradual metamorphosis from a circular configuration to a triangular one aptly exemplifies a realistic progression. Consequently, we may contextualize parallel attraction as a distinctive manifestation within the broader conceptual framework of contour attraction.

### Contour Attraction, Parallel Attraction, or Shape?

In both Experiment 1, which served as the condition for contour attraction, and Experiment 2, designed to elicit parallel attraction, the pairwise comparison of regular octagons and regular hexagons yielded nonsignificant differences, as previously discussed. However, distinct effects were observed for the square and the equilateral triangle, confirming the presence of stronger interaction in these particular polygonal shapes. In Experiments 2 and 3, as well as in Experiments 1 and 3, both the square and the equilateral triangle exhibited longer parallel lines, thereby facilitating pronounced interaction effects. Consequently, the illusion magnitudes associated with these shapes were notably greater compared to those documented in Experiment 1.

In Experiment 2, a progressive transition of the shape occurred from a regular hexagon to an equilateral triangle, leading to a gradual decrease in the distances between the inducers and the test figures. In Experiment 3, the areas of the inducer and the distances between the test figure and the inducer in the regular polygons were matched to those of the circle, and all dimensions were larger than the corresponding shape areas in Experiment 2. The illusion magnitude for the equilateral triangle in Experiment 3 significantly surpassed that of Experiment 2, indicating the impact of distance-induced overestimation, a manifestation of contour attraction. Nevertheless, this effect was weaker compared to the influence of contour attraction observed in the square. When parallel lines are extended, they create an optimal environment for parallel attraction, thereby enhancing the strength of the attraction. Consequently, in shapes that feature elongated parallel lines, the interaction manifested as parallel attraction exerts a more pronounced influence.

Trend analysis of illusion magnitude in Experiment 1 revealed a gradual decline from the regular octagon to the equilateral triangle, with no discernible inflection point. However, Experiment 2 showed a different form of this pattern, as illusion magnitude did not decrease uniformly across shapes; in particular, the square emerged as an inflection point, marking the transition from contour to parallel attraction. In Experiment 3, subsequent adjustments for variations in area and distance between the test and inducer accentuated the effects of parallel attraction, with the regular hexagon now assuming the role of inflection point. The trend lines observed across the three experiments suggest a dynamic interplay characterized by interconversion.

As the spatial frequency of the stimulus pattern increases, the change in shapes correlates with alterations in the size of receptive fields, thereby resulting in either an increase or decrease in the overlap of neural responses to the edges of the circle. According to Moutsiana et al. (2016), such changes in overlap correspondingly entail a decrease or increase in the distance between the peaks of the population response, consequently leading to a decrease or increase in perceived size (Kirsch & Kunde, 2021). This observation elucidates the microscopic mechanisms underlying the illusion phenomenon, while also providing a rational explanation for contour attraction at the macroscopic level of shape perception.

Therefore, our proposition posits that the pivotal determinant influencing the magnitude of the illusion lies in the shape of the inducers. The observed variations in illusion intensity consistently correlate with alterations in either the configuration of the inducers or the test figure. These modifications instigate a transformation in the expression of contour attraction, transitioning between different forms, notably parallel attraction.

Overall, our study makes contributions to advancing the understanding of the Delboeuf illusion. Although some researchers have hypothesized that contour attraction and parallel attraction may transform into one another, there has been a lack of supporting data. Our research empirically supports the notion that these two types of attraction are, in fact, different forms of a single kind of attraction. This finding provides an empirical foundation for this theoretical claim. Illusions are notably complex phenomena, with shape playing a crucial role in their recognition. Our study underscores the importance of considering the influence of shape when evaluating illusions. We present compelling evidence from a novel perspective that shape significantly affects the perception of illusions. These findings contribute to advancing our understanding of how contextual factors surrounding an object can deceive our perception of its size.

### Limitations

Prior investigations have consistently demonstrated a perceptual phenomenon termed the “oblique effect,” wherein individuals exhibit superior accuracy in judging cardinal orientations (i.e., horizontal and vertical lines at 90° and 180° angles) compared to oblique orientations (i.e., lines at 45° and 135° angles); this phenomenon has been attributed to the uneven neural representation of orientations within the visual cortex (Furmanski & Engel, 2000). In our experiments, when the inducers were a regular octagon, a regular hexagon, and an equilateral triangle, some of their lines were inevitably tilted (not horizontal or vertical), and thus, the visual cortex was less activated; as such, the illusion magnitude would be affected to a certain extent (Deng et al., 2013; Fermüller & Malm, 2004; Mannion et al., 2010; Schwarzkopf & Rees, 2013; Sugihara, 2014). Therefore, the present design should be improved, and rotating the test with respect to the inducer may be an effective solution.

There is no clear boundary between the overestimated and underestimated magnitudes of the Delboeuf illusion. In our experiments, we maintained the ratios of the sizes of the test figures and the inducers between 2:3 and 2:5 to ensure overestimation rather than underestimation occurred. While some illusion magnitudes may be measured for both attractive (overestimated) and contrasting (underestimated) effects of the inducer, it is important to note that this phenomenon is not absolute. It would have been more precise to distinguish between contour attraction and parallel attraction based on statistical metrics of the configuration during the parametric manipulation of shape, rather than relying solely on the similarity of patterns to a circle.

Moreover, in Experiment 3, we maintained equal areas for all the inducers, as well as equal distances between the inducers and test figures, to prevent confounding effects arising from differences in distances and areas. Consequently, the sizes of these inducers were all slightly larger than those used in Experiments 1 and 2. Although both the inducers of the targets and the probe underwent synchronous changes, this might potentially lead to inaccuracies in the final results. Prior research has demonstrated that an increase in the spatial extent of the stimulus can influence the Ebbinghaus illusion (Kirsch & Kunde, 2021); in our experiments, it is not feasible to maintain equal spatial extent while keeping distances and areas consistent across different shapes. Furthermore, with the five shapes used in our experiments, achieving equal spatial extent is almost impossible. Among the contour attraction and parallel attraction effects examined in our study, distance and area are critical factors, and synchronous changes in both the inducers and the probe will help reduce the inaccuracy in spatial extent changes to some extent. Consequently, we have focused on restricting these two factors. This limitation may result in inaccuracies in our findings. Different shapes indeed contribute to differences in spatial extent, and the observed variations in spatial extent can be attributed to the influence of shape. This limitation in spatial extent is something we aim to address in future research.

Furthermore, to systematically modulate the shape of the test and inducer, we chose five shapes as independent variables. These shapes represent five distinct levels, with each level progressively deviating further from a circular form. However, it is important to note that the results show small differences across shapes that do not follow a clear parametric pattern, indicating a lack of systematic change in illusion magnitude across the parametric change in inducer shape. Enhancing the precision of measurements and further refining the methodology are crucial steps for future studies, particularly to mitigate the above limitations.

### Conclusion

The present research focused on studying the Delboeuf illusion using polygons as stimuli, leading to several noteworthy findings. First, the illusion's magnitude was significantly influenced by the shape of the inducer and the test figure. Second, the impact of shape on the illusion's magnitude was mediated by contour attraction mechanisms. Thirdly, configurations with circles or shapes resembling circles exhibit the form of contour attraction, while configurations with shapes possessing longer parallel lines shift toward parallel attraction, both types of attraction enhance the perceived magnitude of the Delboeuf illusion. It is important to underscore that this represents merely one conceivable explanation and by no means the sole mechanism accounting for the impact of shape on the Delboeuf illusion, further research is imperative to identify a more fitting explanation. These findings contribute to the advancement of our comprehension regarding how the contextual factors surrounding an object can deceive our perception of its size.

## APPENDIX

*The forming process of the configuration of a quasi-Delboeuf illusion, using a regular hexagon as an example.*

In this variant of the quasi-Delboeuf illusion configuration, the conventional inducer element is substituted by a regular hexagon. This novel configuration was employed in Experiment 1, while in Experiments 2 and 3, it replaced both the test figure and the inducer concurrently within the traditional Delboeuf illusion arrangement. In Experiments 1 and 2, the test figure or the inducer was utilized as an encompassing **circumscribed circle** for regulating the dimensions of the regular hexagon. In Experiment 3, the areas of inducers were equal in different shapes, and the distances between the inducers and test figures were also equal, so the test circle and the inducing circle were used as **inscribed circles** to ensure that the distances were equal. Across all three experiments, the measurement of the hexagon's radius was aligned with that of a circle. The internal polygon was designated as the test figure, while the exterior polygon assumed the role of the inducer, mirroring the nomenclature of the classical Delboeuf illusion paradigm.
